# Choosing internet-based treatment for problematic alcohol use—why, when and how? Users’ experiences of treatment online

**DOI:** 10.1186/s13722-020-00196-5

**Published:** 2020-06-29

**Authors:** Veronica Ekström, Magnus Johansson

**Affiliations:** 1 The Department of Social Sciences, Ersta Sköndal Bräcke University College, Stockholm, Sweden; 2 Department of Public Health Sciences, Karolinska Institutet, Riddargatan 1 – Mottagningen För Alkohol Och Hälsa, Riddargatan 1, 114 35 Stockholm, Sweden; 3 Stockholm Center for Dependency Disorders, Stockholm Health Care Services, Stockholm County Council, Stockholm, Sweden

**Keywords:** Alcohol use disorder, Substance use disorder, Web-based Intervention, eHealth, Help seeking, Qualitative research

## Abstract

**Background:**

Internet-based treatment has emerged as a cost-effective option for reaching people who for different reasons are not reached by traditional treatment. Internet-based treatment for problematic alcohol use, specifically, has been found to show results on par with other forms of treatment. However, in-depth knowledge of users’ experiences is required to understand what works, and what needs further development. The aim of this study is to investigate the help-seeking motives among users of an internet-based service for problematic alcohol use, as well as the users’ experiences of the support available through the service.

**Method:**

The study consists of a thematic analysis of interviews with 38 former users of the internet-based intervention Alkoholhjälpen.

**Results:**

The analysis shows that health and relationship factors, as well as feelings of shame, were important motives for the users’ decisions to reduce their drinking. Availability and anonymity seem to have been important reasons for choosing internet-based support. The different treatment components, i.e. ICBT program, therapist support and discussion forum, were each perceived as helpful by some users but not by others. Treatment components were described as more useful when users were able to personally identify with the content, and when it helped them reflect on their own alcohol consumption.

**Conclusions:**

There are several aspects that are relevant, beyond the comparison between components, if we want to understand what works and for whom in internet-based treatment. Internet-based treatment services should be generous in terms of options for the users.

## Background

Alcohol use is increasing throughout the world and is today one of the major factors leading to premature death and health loss [1, 2]. Most people with problematic alcohol use never seek or receive professional treatment [3, 4]. Common reasons for not seeking professional support are shame, fear of stigmatization and a wish to solve the problem on one’s own [5, 6].

The Internet has started to be seen as a possible platform for treatment of many disorders, including substance use disorders. Internet-based treatments are available in the form of automated self-help programs, guided programs or therapist lead treatments [7] delivered via the internet to computers, tablets or smart-phones. Anonymity and physical distance can be perceived as positive for persons with previous experiences of stigma or shame [8, 9]. According to Vernmark [10], internet-based treatment can also offer unique transparency for patients in regard to their own treatment. Choosing the option of being anonymous can reduce barriers to self-exposure [11]. Patients tend to admit more drug use and more psychiatric symptoms in internet-based treatment compared to face-to-face treatment [12]. Internet-based treatment is also associated with lower costs [11]. Treatment can be delivered immediately to many individuals, irrespective of time and place [13, 14].

Internet-based interventions reach people with problematic alcohol use who are not reached by traditional care facilities [15, 16]. Internet-based treatment can thus bridge a gap for people wanting help to reduce their alcohol consumption [9]. Internet interventions for reducing problematic alcohol use have shown significantly better effects in terms of reduced alcohol consumption compared to various control conditions [17, 18]. Short, internet-based treatment can increase motivation and has been shown to decrease problematic alcohol use [19]. Extended internet alcohol interventions are even more effective than shorter interventions in reducing alcohol consumption, and guided interventions are more effective than unguided ones [ibid.].

## Previous research

Despite over 20 years of research on the effects of internet-based interventions for substance use, very few have investigated the experience of the users of such interventions. The experiences of 18 users of an internet-based self-help program aimed at reducing alcohol consumption have been investigated in a previous study. The results showed that the perceived privacy of the internet was important in searching for help, and in avoiding stigma and embarrassment [9]. In written feedback during the development of the internet-based self-help program, early users expressed appreciation of the non-judgmental tone of texts and self-help exercises [20]. In focus groups with college students regarding perceptions toward using mobile-health interventions to prevent high-risk drinking, the students expressed that drink-tracking and notifications were useful features [21]. Interviews with 31 members of an online mutual aid group reveal that such groups are a viable alternative for people who encounter barriers when trying to access traditional services. Such groups may serve as a place for people to explore their relationship with alcohol at early stages of change and give them the opportunity to construct and adjust their identity in relation to their problematic alcohol use [22]. Focus-group interviews with therapists who work with internet-based treatment of substance use disorders in Sweden reveal that these therapists identified the experience of time and pace as different compared to face-to-face treatment [23].

## Alkoholhjälpen

Alkoholhjälpen can be described as an internet-based self-help service for reducing problematic alcohol use (which in this article serves as an umbrella term for hazardous or harmful use, alcohol use disorder and alcohol dependence). In Sweden 15% of men and 12% of women in the adult population is estimated to have hazardous alcohol use [24]. Regular treatment for alcohol use disorders are offered for all Swedes at low cost via health care or social services. The internet-based service at Alkoholhjälpen is funded by the public health authorities and free of charge for the users. The service includes an online discussion forum, facts pages, information on where to find face-to-face treatment, an Internet delivered cognitive behavioral therapy (ICBT) program and written asynchronous support from a therapist online. The ICBT program has been evaluated in several studies [25–28]. The content is based on Motivational Interviewing and cognitive behavioral therapy and consists of five main modules (Motivation and feedback on assessment, Drinking goal and self-control, Behavioral analysis of drinking and risk situations, General problem-solving, Preventing relapse). In addition, there are three optional problem-solving modules (Handling feelings, Drink-refusal skills and Handling cravings) and 11 fact sheets (Blood alcohol level, Anxiety, Depression, Anger, Stress, Handling thoughts, Relaxation, Sleep, Leisure activities and Communication). Users are also encouraged to register their use of alcohol and alcohol cravings in a calendar. The program has in previous research been shown to reduce alcohol consumption among its users [25, 28]. Treatment results improve when the program is combined with online support from a counselor/therapist^1^ [26].

There is only one previous study where users of internet-based treatment for problematic alcohol use have been interviewed. The current study is the first one to compare users from the same intervention on the basis of the level of treatment received (including therapist guidance). Studying experiences of users can deepen our understanding of what components are helpful and why, which is essential for developing treatment and services for people with problematic alcohol use.

The aim of this study is to investigate the motives for choosing treatment among users of internet-based treatment for problematic alcohol use and their experience of support from the Alkoholhjälpen. The following research questions will be addressed in the article:

1. What are the motives for choosing internet-based treatment?
2. What aspects of the internet-based treatment do users attribute to changing their alcohol use?

## Method

This study has a qualitative design, using thematic analysis [29]. 38 interviews were conducted with former users of Alkoholhjälpen. The users were recruited among the 1169 participants in the quantitative evaluation of the intervention. Participants were originally assigned to one of three groups:

- Therapist-guided: an ICBT program with online guidance from a therapist (a),
- Self-help: an ICBT program as self-help (b),
- Control: Information on consequences of alcohol use to health and well-being (c).

In addition, all three groups had access to a discussion forum online. Individuals were included if they met three or more ICD-10 alcohol dependence criteria or had a total score of 15 points or more on the Alcohol Use Disorders Identification Test (AUDIT). To be able to complete the registration, the participants needed to understand written Swedish and be computer literate enough to access and navigate the website via a computer, tablet or smartphone [30].

All interviews were conducted by phone by author VE, between May and June 2018. All participants had received written information about the study and ethical considerations. Consent was digitally recorded in the beginning of the interviews. The interviews followed a semi-structured interview guide. The structure was the same for all participants, with some differences depending on intervention group. They were asked to describe how their alcohol consumption had affected their lives, why they had used Alkoholhjälpen, if they had tried other forms of treatment, how they worked with the different parts of Alkoholhjälpen and if/how Alkoholhjälpen had made any difference for them. They were also asked about their present alcohol consumption and if they thought they needed more treatment. Follow-up questions were formulated during the interviews if needed.

Interviews lasted between 20 and 45 min and were digitally recorded. They have been transcribed verbatim by professional transcribers. Quotes have been carefully modified to increase readability. The interviews were made in Swedish and quotes have been translated for this article.

The interviews were read by both authors and were then coded by author VE in NVivo following Braun and Clarke’s guidelines for thematic analysis [29]. The analysis was inductive, and the codes used were developed during the coding and from what the interviewees talked about. The next step was to process all developed codes and merge some of them. Finally, we searched for themes and sub-themes among the identified codes.

To facilitate the assessment of this study’s credibility, a significant number of quotes are presented to increase transparency in the analysis. Quotes are chosen because they are the most vivid examples or the most illustrative in capturing the essence of a certain topic [29]. Recommendations by Braun and Clarke have been followed, which include verbatim transcription, thorough coding and moving back and forth between different parts of the empirical material. A preliminary analysis was presented at a seminar with three experienced therapists from Alkoholhjälpen.

The study follows what is stated in the Act concerning the Ethical Review of Research Involving Humans (SFS 2003:460). It has been approved by the regional ethics committee (Dnr 2017/1660-32). The participants were informed of the study’s purpose and methods, and that their participation would be confidential and voluntary. To protect their identities, some details of their stories have been changed or edited out. All names in the transcripts are fictitious. Records of the interviews were deleted after the interviews had been transcribed. All interviewees received two movie tickets as a gesture of appreciation for their participation.

## Results

In relation to the first research question about motives for choosing internet-based treatment, three major themes were identified, and the table below shows the three themes and their sub-themes (Table 1).

**Table 1:** Motives for choosing internet-based treatment, major themes and sub-themes

The decision to reduce alcohol	Consequences of alcohol use	Reasons for choosing Alkoholhjälpen
Escalation over time	Feelings of regret/shame	Availability
Medical or physical problems	Affects relationships	Anonymity
Felt bad/shame	Risky behavior	Instead of other forms
Relationships	Overweight	No alternatives
Work related	Fatigue	
Curious/as an experiment	No consequences	
No special reason		

Our second research question focus on different parts of the support provided by Alkoholhjälpen. We identified thirteen sub-themes which we have sorted under three major themes: (Table 2).

**Table 2:** Experiences of Alkoholhjälpen, major themes and sub-themes

ICBT-program	Discussion forum	Therapist guidance
Identification	Helpful	Important
Reflection on alcohol use	Identification	Useful
Finding patterns in alcohol use	Support from others	Missed personal contact
Written material	No confidentiality (negative)	
	No identification (negative)	
	Not interested	

### Participants

All 38 users who wanted to participate were interviewed. 18 were female and 20 were male, and all were between 26 and 74 years old. See Table 3 for details.

**Table 3:** Age of the participants

Age	25–34	35–44	45–54	55–64	65–75
N	2	6	14	8	8
Percentage	5%	16%	37%	21%	21%

Participants were recruited from all three groups from the original evaluation, described in Table 4 below. During the interviews, three participants from group c revealed that they had later participated in the therapist-guided program.

**Table 4:** Number of participants from each group

Gr	Intervention group	N
a	Therapist-guided ICBT program and discussion forum	20
b	Self-help ICBT program and discussion forum	9
c	Information and discussion forum	6
c + a	Information, discussion forum and later therapist-guided ICBT program	3
	Total	38

### Alcohol use—past and present

Most of the participants describe various forms of negative consequences from alcohol. Those who do describe negative consequences all talk, to a varying degree, about how being tired or hung over has affected them. Some have exposed themselves to risks or “done stupid things” while being drunk, and some feel regret and shame. Several describe consequences in term of negative impact on relationships, for example, children being affected and risk of divorce, or even having already lost a partner. Feelings of regret and shame are more often described by women and it is only women who mention gaining weight as a negative consequence of alcohol.

Most participants had not sought treatment for problematic alcohol use before Alkoholhjälpen. However, several of them describe having tried to reduce their drinking on their own. Some have previous experience of different health care facilities or pharmaceutical treatment. Alcoholics Anonymous (AA) is also mentioned by a few. Approximately half of the participants say they are now pleased with their present alcohol consumption. The other half of the participants describe their alcohol use as still being problematic.

### The decision to reduce alcohol use

Some of the participants describe relationship problems as a factor contributing to their decision to try treatment. For example, Jörgen says his girlfriend demanded that he stop drinking, and Jens says starting treatment was a way to save his relationship. In the quote below, Anne describes her decision to start treatment via Alkoholhjälpen:

I had to process something within myself. Because I got that question from both my family and my husband. Why? Why have you tried to hurt yourself and us in this way? And I couldn’t answer that. I had no clue. (Anne, female, age 33).

For Anne, her family was a contributing factor to her decision to change her situation. Relationships and change are also described by some of the other participants in terms of wanting to change their situation after a breakup.

For Irene and Iris, it was the feeling of shame that made them decide to change their alcohol use. Irene says she felt mentally ill and was ashamed. Iris talks about a party when she lost control as a decisive moment:

It wasn’t a problem at all for many years…I was aware of this and I was really trying to think about what I drank and how much. Especially when I went out. You’re in more control when you’re at home. (…) And then, I went to a party and it got too much and I just felt that…no. I’ve felt that many times, but now it had to end. Now it’s enough and I don’t want to put myself through this anymore. (Iris, female age 52).

Magdalena was told she had a poor liver, and for her that was a signal that she needed to change her drinking habits. Ulf, Mats and Tim talk about friends or relatives who had recently been affected by alcohol-related injuries, which made them think about their own alcohol use. However, the most frequent observation among the interviewed participants is that nothing extraordinary has happened. Rather, they describe a sense of having reached a point where they were drinking too much or too often. Several say they had thought about reducing alcohol consumption for a couple of years. One example is Håkan, and this is how he describes it:

I think it was an ordinary Monday when I simply…I’ve had that feeling on many Mondays. (…). It’s not like I can speak openly about it, but realized I’ve got some sort of alcohol problem many years ago. It has been quite obvious to me. (Håkan, male, age 45).

### Why Alkoholhjälpen?

For most of the participants, nothing special had happened that made them decide they wanted to reduce their drinking and that they needed help to do so. Choosing Alkoholhjälpen is also not described as a deliberate decision. The participants say they “googled” or searched randomly on the internet and found Alkoholhjälpen. Some of them say availability was important, i.e. being able to make contact when they needed. Frida’s story is an example of this, and in the quote below she answers a question on why she chose Alkoholhjälpen and whether she could just as well have gone to a health care facility, a clinic for alcohol treatment or the social services:

No. I’m an extremely early riser and there is nowhere…I’m up at 05:20. That’s my time for reflection (…) and there are no open clinics at that time. I started googling, found it and thought I should give it a try. (Frida, female, age 50).

Mikael describes why it happened to be Alkoholhjälpen in similar ways as Frida. It suited his irregular working hours. Sven says he cannot be absent from work during working hours and Pia, who has thought about AA, has problems finding a babysitter. The availability of internet-based treatment suits them better. Others say they did not want group treatment, wanted to avoid religious influences as in AA, or did not want personal contact with a therapist. Birgitta says she chose Alkoholhjälpen because it was available, and because she could avoid being watched, thus not needing to think about what clothes she was wearing.

Several of the participants say it was important to be able to be anonymous, especially initially. It made it possible to be more honest in their answers. But for some participants, anonymity has not been important at all. Some of the participants who are parents, say they have deliberately chosen treatment outside the social services or public health care due to a concern about what could happen if their problematic alcohol use became known. Pia in the quote below exemplifies this:

As a parent and a single mother…I mean, it just can’t get out. It’s as simple as that. No one can know because I could lose my child and that’s not a risk you’re willing to take. Not me at least. And what if I told the doctor or something and they took my child immediately…what if I didn’t even get a chance to….sure, I understand it might not be like that, but the thought of it, that it could happen…you don’t go there. I won’t. Therefore, I needed something where you could be anonymous. (Pia, female, age 39).

Emanuel has similar experiences. He searched for professional support where he lives and was referred to the social services. However, this was an unthinkable option for him. He was not willing to risk the involvement of the social services:

I simply couldn’t risk having the social services interfering with my relationship with my children. The social services clinic for alcohol dependency was not an option for that reason. (Emanuel, male, age 52).

Anonymity can be about not being recognized, and/or not having to be open about your difficulties. Anonymity can also be a protection against the authorities, as Pia in the quote above describes. Anonymity can also lead to increased autonomy, which Håkan gives an example of:

What you can feel if you go to traditional public health care or similar is that it is taking on more of a police style. You’re sort of afraid that you’ll get caught in some sort of yarn where you’re not the one in control. (Håkan, male, age 45)).

Anne went to her occupational health care services and met a nurse. She thought it was a good meeting, but to continue in treatment for her problematic alcohol use, she had to tell her employer. For her, it was unthinkable.

It was very difficult for me then. That it was demanded so early. You’re not only going to quit drinking and try helping yourself, but you must also…I mean, it’s an embarrassing disease. Or it’s an embarrassing problem. You don’t want to share it with the whole world. That’s how I felt. And I felt like I won’t do it. (Anne, female, age 33).

### The program

The following section is based on only those participants who have either been part of the therapist-guided group (ICBT program with online guidance from a therapist) or the self-help group (ICBT program, as self-help), since the control group have not had access to the program. The time the participants have worked with the program varies. Many of them say 3 months, but shorter periods such as 4 weeks are mentioned. Some of the participants are critical of the three-month time limit of counseling, and say they would have needed more time.

Birgitta is one of the participants that has had support from a therapist when following the program. She was pleased with her present alcohol consumption at the time of the interview. Her description of how she worked with the program can be interpreted as an example of how internet-based treatment works when it works well:

I felt motivated all the time and I thought it was a quite challenging task, really. When the day started to come to an end, I sat down and started to…sort of evaluate and go through things. What does the program say? What has happened? What do I feel? And so on. I really took that time every day. It was great to have that demand on you, that I should do it. (Birgitta, female, age 68).

Another participant who has had support from a therapist is Anne. She says she has got explanations for her own behavior through the program and she has recognized herself in the texts:

I remember those texts (…) I can’t mention any typical example, but I remember that when I sat down and read them, I felt like “aha, that’s why I react like I do or behave as I do”. I remember there was a lot of recognition in those texts. (Anne, female, age 33).

The sense of identification is mentioned in several interviews. What Anne describes in the quote above is also how the program has helped her to reflect on her alcohol consumption. It is an aspect that several participants mention as important. Reading texts and answering questions help them to reflect. Marie is an example of this. She belongs to the self-help group. She describes the benefits of the questions in the program and that these led to thoughts she might not have had otherwise:

The questions make you reflect. Reflect on things that you might not reflect on otherwise. I can’t remember any example or any specific question, but I know I had thoughts in my head that I might not have had otherwise. (Marie, female, age 46).

While the feeling of recognition is described as an important positive aspect of the program by some participants, other say the lack of recognition in the material has been a barrier. Sven belongs to the therapist-supported group and he says the “tools and booklets” as he calls the program, contained much repetition and he thought it was hard to know what to learn. He describes the questions as unhelpful since they were not written for people like himself, who wants to quit drinking completely. This is also an example of how the lack of identification can be a barrier to treatment.

Several of the participants say the drink-tracking function in the calendar has been important. By using the calendar on a regular basis, they have been able to see patterns in their drinking behaviors. Irene is an example of a participant who emphasizes the importance of the calendar function:

If I remember it right, I registered situations. I think it was people I had met and quite straight-forward…like how much have you had to drink this day or this week? You registered. Tools like that work very well for me. To sort of specify and map and see a pattern in a way I perhaps had not been able to see just by trying by myself. It was very concrete. (Irene, female, age 53).

The participants have been able to choose when and where they want to work with the program. They can control the pace, which for some has been an important aspect. Even though it is possible to go back and read old material and tasks in the program, few mention having done so. Some say they tried to, but that they found it difficult to locate the material. One of the participants suggests the program should include a summary of what has been completed and several others suggest e-mails to remind you of Alkoholhjälpen after finishing.

Alkoholhjälpen has, for some of the participants, been a stepping stone to other treatments. Some of them say Alkoholhjälpen has helped them to come to a decision concerning their alcohol consumption and to realize they needed more support. For example, Henrik says the program has been important for being able to take the step to reduce his drinking. Hannes says the program did not help him in any other way than getting closer to a decision. Jens has a similar description:

The tracking helps you to put this puzzle together and come to a decision. To do something. Get yourself together. To stop sliding like a glacier. (Jens, male, age 57)).

There are also participants who are less satisfied with the program. One criticism is that there are too many texts, and that they are too long. Some talk about repetitive material and that it is boring to answer questionnaires and write down reflections. One example of this is Harriet. She says she got tired and thought the program was difficult. In the quote below, she is asked to describe in what way it was boring and difficult:

It was sort of interesting at the beginning. I was supposed to fill in forms every week. A bit like Weight Watchers. Exactly how much I had been drinking. (…) And I got questionnaires I should answer. I got some response and that was kind of nice. But then, nothing else happened. It was sort of like that for me. It didn’t change my habits. I didn’t get more attentive. Rather, when you answer questions, you tend to underrate your consumption. It’s easy to lie. (Harriet, female, age 60).

### Therapist guidance

Some of the participants from the two study groups without support from a therapist talk about this as something they have missed. Many of the participants who have received support from a therapist during their treatment at Alkoholhjälpen, are positive about the support they have received. They seem to perceive the support as a personally meaningful. One example of how this is expressed is the following quote from Jens:

I think it was positive because it’s a living person at the other end…I felt it was that. That someone was there, someone was watching. It was probably not a computer program, but real people who were behind this and that you should have contact with. (Jens, male, age 57).

Johan is another participant who has appreciated therapist feedback. He says it has been helpful to have another person’s opinion. Others talk about the importance of having someone who cared or who supervised their work. This is expressed by Tom as follows:

I looked forward to getting the response when I had completed different assignments. (…) It felt very genuine and honest somehow. Yes, like someone cared. (…) She helped me and had reflections on this and what she thought and…yes, I thought it was professional. I mean, I could tell that she was good at her work and she had probably worked for many years with this kind of problem. (Tom, male, age 59).

Those who have received support but are less positive mostly talk about meaningless feedback. For example, Sven who received feedback a few times, but felt it did not mean much to him:

I got it (feedback, my remark), but what was actually said…I really can’t remember. But it was extremely little. Didn’t do anything for me. No. The memories are a bit blurred. (Sven, male, age 55).

Sven continues by saying he would have liked more support or feedback, and that recurs in several of the interviews, e.g. the interview with Maria. She has had support from a therapist, but would have liked a closer relation with a therapist and more guidance in the program:

I fool myself all the time so perhaps that’s what I’m missing. I complete a lot of forms and stuff but try to help myself to get to the bottom of things! (…) I think the concept [of Alkohohjälpen] is really good, but it’s up to me if I want feedback or not…I mean, it’s up to me all the time. There’s no one to hook me up. And I think I need that. (Maria, female, age 57).

### The discussion forum

The open discussion forum is the one component that all participants in this study have had access to. Also, as described earlier, it has been the only component for participants in group c. Just over half of the participants describe the discussion forum as useful. Such participants are found in all three study groups. Based on the interviews in this study, there are no patterns indicating that access to other components of Alkoholhjälpen influences the users’ opinions regarding the discussion forum.

Some of the participants talk about feeling uncomfortable talking about their problems in public and therefore are worried that they would be identified in the discussion forum, despite the anonymous aliases people use in the forum. Some participants are not comfortable about speaking about their problems with others—no matter if it is in public or not. Others say they are not interested in others’ experiences or that it is too energy-consuming to read about other people’s problems. Those who are more negative talk about not feeling a sense of belonging with others in the discussion forum. Several say they think that those who were active in the discussion forum had much more severe problems than themselves.

However, reading about other people’s problems is also described as a positive aspect by several participants. For example, Irene says it has been useful for her to read other people’s stories. It released some of the shame, she says. Irene also talks about the positive effect of reading about others doing well, something that is also mentioned by other participants. Recognizing that you are not alone is another helpful aspect of the forum that recurs in the interviews. For some of the participants, it has been useful to realize that others have similar problems to them. Klas is an example of this. He belonged to the therapist-guided group who had access to the program, therapist support, and the discussion forum:

I’ve read and I’ve written. I feel I’ve made contact with different people who have given me a lot there. Yes, that’s how it is. I actually think it helps me to stay sober by relating to others with similar problems as mine. (Klas, male, age 74).

Emanuel also belonged to the therapist-guided group, and for him the discussion forum was the most important component and the thing he used the most. He still struggles with his problematic alcohol use. He is quite critical of Alkoholhjälpen, but talks about the discussion forum in more positive ways. The quote below is his answer to a question on whether any parts of Alkoholhjälpen have been helpful:

I must say very little has helped. The thing that has been some kind of help was this discussion forum. That you could feel some sort of support from real people. (Emanuel, male, age 52).

Johan was also part of the therapist-guided group and he is one of the participants who did not drink alcohol at all at the time of the interview. His experiences are similar to Emanuel’s:

What works for me with Alkoholhjälpen…it was most of all the discussion forum. The best thing was that there were other people there. It helped. I didn’t write anything, I just read. All the people’s comments and what they had been through and suffered from. You sort of felt you weren’t completely useless after all. There are others out there with problems. (Johan, male, age 39).

## Discussion

The aim of this study was to investigate the motives for choosing internet-based treatment among users of an internet-based intervention for problematic alcohol use, and their experiences of the treatment received. In summary, the analysis revealed that health and relationship factors, and feelings of shame, were important motives for the users’ decision to reduce their drinking. Availability and anonymity seem to have been important reasons for choosing internet-based support. The different treatment components, i.e. ICBT program, therapist support and discussion forum, were each perceived as helpful by some users but not by others. Treatment components were described as more useful when users were able to personally identify with the content, and when it helped them reflect on their own alcohol consumption.

Regarding why they chose to start using internet-based support, only a few of the participants say something extraordinary had happened or that they had an epiphany of some kind. For most of the participants in this study the answer is that there was no specific reason. Rather, it is described as a coincidence that they found Alkoholhjälpen when they searched the internet. As the analysis shows, most of the participants said that they had increased their alcohol consumption over time, to a level they were not pleased with. Participating in Alkoholhjälpen could for them be interpreted more in terms of exploring their concerns with their alcohol consumption rather than actively searching for treatment. This is similar to the views on internet-based treatment expressed by non-treatment seekers with alcohol dependence in a focus group study [6].

Anonymity and availability of treatment have been identified in previous research as important motives for choosing internet-based treatment [11, 31]. Availability is mentioned in several of the interviews, both in terms of having trouble accessing traditional treatment due to geographical reasons, but more often in terms of access to treatment being limited by the opening hours of clinics or care facilities. Asynchronous internet-based treatment like Alkoholhjälpen enables treatment when it suits the users. They can work with the program when they want and do not have to make special arrangements, such as taking time off work or hiring a babysitter. As for the importance of anonymity, feelings of shame and stigma are still barriers for treatment, and some of the participants say they would never have started treatment if they had been obliged to go to the social services or occupational health care. Fear of having your parental abilities questioned and investigated by the social services is mentioned by several of the participants. Being able to choose anonymous treatment outside the social services has been crucial for them.

For some participants in this study, Alkoholhjälpen has also served as an alternative source of treatment where you can get support for treating less severe problems. If you do not define yourself as a person with a severe problem, you might not think you are part of the target group, or even doubt that you will be allowed traditional forms of treatment. These descriptions support previous research that has shown that internet-based treatment reaches people who are not reached by or interested in traditional treatment. [15]. These results are also in line with those found in previous interviews with users of an internet intervention for alcohol [9].

The second research question was what role the users attribute to different parts of the support provided by Alkoholhjälpen in changing their alcohol use. The interviews in this study were conducted approximately 2 years after the participants had completed the program. Several of them found it hard to remember what the program consisted of. It is obvious that many do not seem to remember details of the program. Therapists at Alkoholhjälpen say that the written form and technical design may enable users to go back to their material on several occasions in internet-based treatment [23]. This function does not seem to have been utilized by the participants in this study. Some of them suggested that more reminders, in form of e-mails, for example, would perhaps increase re-visiting of the written material—both the psycho-educative texts and the users’ own reflections. The helpfulness of the drink-tracking function included in the program’s calendar is emphasized by several participants. Tracking alcohol consumption and getting feedback have been identified in previous research as an important component of internet-based interventions [21, 32].

Previous studies have shown that therapist guidance increases treatment effects [19, 26]. The results in this study indicate that therapist guidance is important or even vital for some participants and not necessary at all for others [33]. The guidance through feedback has been perceived by some participants as shallow or meaningless. There is a challenge in providing the right amount and type of guidance to each participant since the participants have such varied needs of guidance.

The discussion forum is described as having two functions. It is an interactive forum where you can meet others and discuss different aspects of your problematic use of alcohol, your struggles and your successes. But some users choose to just read and find inspiration in the life stories of others, without posting content of their own. This is similar to the findings of Chambers [22] where most participants reported spending time with consuming already available content, before taking an active role in discussions. The level of interaction users choose in discussion forums for problematic alcohol use is not particularly well-explored in previous research and represent an important topic to address in future studies.

The special form and pace of internet-based treatment in written form seems to have some advantages. The users do not need to answer immediately, as is the case with oral conversation. It is possible to take time to think before formulating a response or completing a task in the program. These results support previous findings on the advantages of online communication [34]. However, even though Alkoholhjälpen is easy to access and is described as available in many ways, the components can be difficult to use for some. It best suits people who are used to reading and writing and feel comfortable doing so.

Since this study uses a qualitative design, generalizing results in the same way you would when using a quantitative method is not possible or intended. These participants are not supposed to represent all users of Alkoholhjälpen or users of internet-based treatment. However, their experiences can be used to deepen the understanding of and knowledge about internet-based treatment for problematic alcohol use in general, and Alkoholhjälpen in particular. The results are discussed in relation to previous research in the final part of the article, which places the study in a wider context.

## Conclusion

This study shows that the users’ perspectives on internet-based treatment for problematic alcohol use are complex. Participants have different ideas about what has worked and what has been useful for them. Text-based self-help treatment is appreciated by several users while the personal contact and feedback from a therapist is central for others. For some, it is tracking alcohol consumption in a calendar that is described as the most important aspect, and others say interacting with others or reading about others’ problems in a discussion forum has been the most useful. This complexity and variation needs to be considered, beyond a comparison between internet-based ICBT programs and other components, if we want to understand what works and for whom in internet-based treatment. Availability in the form that internet-based services like Alkoholhjälpen provide, enables more people to get treatment for their problematic alcohol use, including those who are not reached by traditional face-to-face services. Further development of internet-based interventions for problematic alcohol use is important, both as an alternative and a complement to traditional forms of treatment.

## Data Availability

The qualitative data analyzed during the current study are not publicly available to protect the participants’ identities. The transcribed interviews are in Swedish. Translated pseudonymized data are available from the corresponding author on reasonable request.

## Abbreviations

ICBT: Internet delivered cognitive behavioral therapy
AA: Alcoholics Anonymous

## Footnotes

1 Guidance is provided by experienced staff with an educational background within psychology, social work or behavioral science.
